# Assessment of Gait Disorders in Cerebral Small Vessel Disease: Advantages of Different Clinical Scales

**DOI:** 10.3390/jcm14186626

**Published:** 2025-09-19

**Authors:** Larisa A. Dobrynina, Elina T. Bitsieva, Kamila V. Shamtieva, Maryam R. Zabitova, Marina V. Krotenkova

**Affiliations:** Russian Center of Neurology and Neurosciences, 125367 Moscow, Russia; dobrla@mail.ru (L.A.D.); m_zabitova@mail.ru (M.R.Z.);

**Keywords:** cerebral small vessel disease, gait disorders, clinical scale, risk of falls, Tinetti test

## Abstract

**Background/Objectives:** Cerebral small vessel disease (cSVD) is one of the leading causes of gait disorders (GDs) in the elderly. Clinical diversity and lack of standardization in assessment of GDs in cSVD patients are associated with late diagnosis. The comparative value of clinical rating scales used for gait assessment in clinical studies of cSVD has not been previously clarified. The purpose of the study was to assess GDs in cSVD patients with different scales and evaluate the advantages of their usage in clinical practice. **Materials and methods:** The study included 124 cSVD patients (STRIVE, 2013) (average age 62.2 ± 7.9, women—53.2%) and 30 healthy volunteers (average age 59.77 ± 6.361, women—56.7%). Gait and balance function were assessed with the Tinetti test, “6-m walk” test, and the Clinical Scale for Assessing the Severity of Gait Disorders in SVD (RCN, 2019). **Results:** In total, 85 (68.5%) patients had gait disturbances. The “6-MWT” showed a general tendency to decrease gait speed, step length, and increase in base width. ROC analysis established their thresholds for GD diagnosis. Moderate- or high-risk of falls was found in 52 (41.9%) patients. Gait parameters assessed by two tests (Tinneti and 6-WMT) showed a high degree of intercorrelations. Comparative analysis of the quantitative parameters of Tinneti and 6-WMT tests revealed significant differences depending on the severity of the GD assessed by the Clinical Scale for Assessing the Severity of GDs in cSVD (RCN, 2019). **Conclusions:** GDs in cSVD are characterized by slowness, changes in step length, base width, and a high risk of falls. The Tinetti test and “6-MWT” have good reproducibility in cSVD, high correlations between the tests, as well as significant differences between the categories of GD severity, which justifies their use in cSVD patients. The advantage of the Tinetti test is the ability to perform a fall risk assessment, while “6-MWT” allows for the diagnosis of GD based on gait parameter thresholds, which is important in the early stages of the disease and in dynamic observation. The Clinical Scale for Assessing the Severity of Gait Disorders in cSVD is a convenient screening tool for assessing the severity of GDs in clinical practice.

## 1. Introduction

Cerebral small vessel disease (cSVD), associated with age and vascular risk factors, is characterized by pathological changes in small cerebral arteries, arterioles, capillaries and venules, which lead to damage to the white and deep gray matter of the brain and are accompanied by a complex of clinical and neuroimaging manifestations (white matter hyperintensities (WMHs), recent small subcortical infarcts, lacunae, microbleeds, cerebral atrophy, and cortical microinfarcts) [1]. cSVD has a high prevalence in the general population, occurring in 5% of people under 50 years of age and in 100% of people over 90 years of age [2]. In the cSVD development, the initiating role is given to endothelial dysfunction with increased blood-brain barrier permeability, which subsequently leads to structural damage to small vessels and the brain [3].

cSVD is a leading pathology among cerebrovascular disorders and major neurological cause of gait disorders (GD) [4,5,6,7,8].

For a long time, GDs were not regarded as an independent clinical presentation of cSVD and were rarely assessed in large population-based studies.

Recent evidence suggests that GDs are the second most prevalent clinical syndrome in cSVD after cognitive impairment (CI) [9,10,11]. It is also a more sensitive predictor of 8-year mortality than CI, even after adjustment for age, sex, and vascular risk factors [12].

The first descriptions of GDs in cSVD date back nearly a century [13,14]. The heterogeneity of their presentation has led to numerous descriptive terms, based on anatomical localization and/or clinical phenomenology, such as frontal gait apraxia, cerebellar gait, subcortical gait, among others [15]. Even today, researchers acknowledge significant difficulties in differentiating and classifying cSVD-related GDs, as well as in their clinical assessment [16,17], which limits objective disease monitoring and treatment evaluation.

A variety of gait assessment tools have been employed in cSVD studies, including the Tinetti test [18,19,20,21], timed walk tests over a fixed distance (e.g., 6 m) with gait speed calculation [10,22,23,24], the Short Physical Performance Battery [10,25,26], and the “Timed Up and Go” test [20,21], among others.

In 2019, we proposed the “Clinical Scale for Assessing the Severity of Gait Disorders in Cerebral Small vessel Disease” (Patent No. RU2711602C1, 2019). The validity of the proposed severity categories was confirmed by phase-contrast magnetic resonance imaging (MRI), which demonstrated a statistically significant progressive imbalance in brain fluid dynamics, manifested by increased arterial stiffness with reduced compliance, decreased venous outflow with elevated venous pressure, and the consequent development of internal hydrocephalus (enlargement of the aqueduct and lateral ventricles) [27].

The difficulties of early diagnosis and differentiation of GDs in cSVD, the non-specificity of existing tests/scales, and the lack of comparative data for this pathology [11,28,29,30,31,32,33] justify the need to evaluate GDs in a single cSVD patient cohort using the most frequently employed tests/scales and to compare their parameters. This will allow us to establish the correspondence between qualitative scales and quantitative tests and to clarify their advantages for use in clinical practice and scientific research.

### Objective

The purpose of the study was to assess GDs in cSVD patients with different scales and evaluate the advantages of their use in clinical practice.

## 2. Materials and Methods

The study included 124 patients aged 45–75 years with age-related cSVD presenting with MRI signs of the disease according to STRIVE criteria (2013) [3]—white matter hyperintensities (WMHs) of Fazekas grade 2–3—and complaints of GDs and/or CI.

Exclusion criteria: cSVD due to other causes; MRI abnormalities in the brain other than cSVD; gait disturbances resulting from other neurological conditions, such as paresis, hyperkinesia, motor or sensory polyneuropathies with impaired joint position sense, marked pain syndrome, or neuropathic pain; radiculopathy of the lumbosacral spinal nerve roots with motor and pain syndromes; non-neurological causes include myopathies, advanced (grade 3–4) osteoarthritis of the lower limb joints, and severe joint pain syndrome; uncorrected visual impairment preventing completion of the walking distance; atherosclerotic stenosis of extra- or intracranial arteries > 50%; amnestic type of CI due to probable Alzheimer’s disease; severe somatic pathology; and contraindications to MRI.

The control group consisted of 30 volunteers matched for sex and age, without GDs or CI and without MRI evidence of brain pathology. All participants provided written informed consent to participate in the study. The study was conducted in accordance with the Declaration of Helsinki and approved by the Local Ethics Committee of the Russian Center of Neurology and Neurosciences (Moscow, Russia). The ethics statement number is 10-1/22 dated 23 November 2022.

All participants underwent a general clinical evaluation and neurological examination, including assessment of pain and vibration sensitivity as well as joint position sense.

Pain sensation was assessed using a special safe sterile needle and vibration perception—a low-frequency tuning fork (64–128 Hz). The preservation of joint position sense was determined as the patient’s guessing of the direction of passive movements (up-down) in the fingers and toes performed by the physician-researcher.

In cases of clinical symptoms of polyneuropathy, stimulation electroneuromyography was performed. Range of motion in the hip and knee joints was evaluated, and radiography was performed if necessary. All patients were examined by a physician, and if a history of diabetes mellitus was present, the patient was looked after by an endocrinologist from Russian Center of Neurology and Neurosciences.

The severity of CI was evaluated using the Montreal Cognitive Assessment (MoCA) [34] and the scales for independence in daily living [35,36]. The patients were assessed by the following criteria: Subjective CI (subCI)—cognitive complaints and MoCA ≥ 26 points; Mild cognitive impairment (MCI)—MoCA < 26 points with preserved independence in daily living; Dementia—MoCA < 26 points and loss of independence in daily living.

Testing was performed in the early afternoon on a non-slip surface; participants wore their usual footwear with closed toes, a fixed heel counter, and a flat sole; spectacle correction was used in the presence of visual impairment; and they wore loose sports trousers or pants that did not restrict step length, with stable hemodynamic parameters. Patients were instructed on the importance of performing the tests at their usual walking pace.

The GD assessment protocol included several scales and tests:

1. *Performance Oriented Mobility Assessment* (POMA, or Tinetti Test, TT) [15], comprising the “balance” subscale (0–16 points) and the “gait” subscale (0–12 points). Based on the total score, all participants were classified into groups at high (<19 points), moderate (19–23 points), and low (≥24 points) risk of falls [18,37].
2. *Six-Meter Walk Test* (6-MWT, adapted from Tiedemann A. et al., 2008) [38]. Participants were instructed to walk a 6 m distance on a flat carpeted surface. To ensure steady gait speed during the measured 6 m, walking was initiated 1.5 m before the start line (initiation phase) and concluded 1.5 m beyond the end line (deceleration phase). In addition to measuring walking time and speed, a number of additional gait parameters were assessed. Before testing, the soles of participants’ shoes were marked by walking in place on a surface coated with a coloring agent (talc). Gait parameters were assessed using a stopwatch and a measuring tape.
  Parameters evaluated in the 6-MWT are as follows:
  ○ Time to cover the 6 m distance (s);
  ○ Number of steps required to cover 6 m (n);
  ○ Gait speed (m/s);
  ○ Maximum and minimum step length (distance measured in the sagittal plane between the posterior [heel] edges of the right and left feet, cm) [39];
  ○ Maximum and minimum base width (distance measured in the frontal plane between homologous points of the right and left feet, cm) [39].
3. *Clinical Scale for Assessing the Severity of GD in cSVD* (Research Center of Neurology, RCN) [Patent No. RU2711602C1]. Testing involved walking in a straight line for 4–5 m, followed by turning and returning to the starting point. Gait was evaluated both at the usual pace and under more complex conditions (tandem and side walking). The severity category of GDs was determined according to clinical characteristics (Table 1).

The sequence of tests for gait assessment was as follows: (1) Clinical Scale for Assessing the Severity of Gait Disorders in SVD (RCN, 2019); (2) the Tinetti Test; (3) the 6-Meter Walk Test. The study was split over two days: on the first day, a clinical gait assessment was performed alongside a general clinical and neurological examination; on the second day, the Tinetti Test and the 6-Meter Walk Test were conducted. The researchers performing the gait assessments were blinded to the patients’ clinical and MRI data.

All participants underwent brain MRI (3T MR scanners Siemens MAGNETOM Verio [Siemens AG, Erlangen, Germany] or Siemens Magnetom Prisma [Siemens AG, München, Germany], using protocols required for the assessment of MRI signs of cSVD according to the STRIVE criteria.

Statistical analysis was performed using “IBM SPSS Statistics, version 26.0” (IBM Corp., Armonk, NY, EE.UU.). For categorical and ordinal variables, descriptive statistics were presented as frequency and proportion (%); for continuous variables, as mean ± standard deviation (mean ± SD) or median with the 1st and 3rd quartiles (Me [Q 25%; Q 75%]). Two-tailed statistical tests were applied. The null hypothesis was rejected at *p* < 0.05. Comparative analysis of categorical variables was conducted using Pearson’s χ^2^ test or Fisher’s exact test. Correlation between continuous variables was assessed using Pearson’s correlation method, with evaluation of correlation significance. Comparison of continuous variables among three or more groups was performed using the Kruskal-Wallis test. Post hoc pairwise comparisons were also performed using the Kruskal-Wallis test with subsequent Bonferroni correction for multiple testing. Receiver operating characteristic (ROC) analysis was used to determine cut-off values of gait parameters for GD diagnosing.

## 3. Results

The general characteristics of the groups, including demographic data and risk factors, are presented in Table 2.

The control group (n = 30) was comparable to cSVD patients in terms of sex and age. Among 124 cSVD patients (mean age 62.18 ± 7.852 years; women—66 [53.2%]) hypertension, diabetes mellitus, and hypercholesterolemia were significantly more common compared to the control group.

The clinical characteristics of cSVD patients and the analysis of MRI signs are presented in Table 3.

CI was diagnosed in all cSVD patients (mean MoCA score 22.85 ± 4.72): subCI in 44 patients (35.5%), MCI in 47 (37.9%), and dementia in 33 (26.6%).

According to Clinical Scale for Assessing the Severity of GDs in cSVD (RCN, 2019), GDs were identified in 85 cSVD patients (68.5%): mild—in 34 (27.4%); moderate—in 18 (14.5%); severe—in 29 (23.4%), with the frontal-cerebellar subtype in 11 (8.9%) and the frontal-subcortical subtype in 18 (14.5%); and profound—in 4 (3.2%).

Other neurological presentations in patients with cSVD included pseudobulbar syndrome in 35 (28.2%) and pelvic floor dysfunction in 38 (30.6%). In 4 patients (3.2%) of the main group, hip or knee osteoarthritis of grade 1–2 was diagnosed, without joint pain and without an impact on gait; mild sensory polyneuropathy was present in 8 (6.5%) patients, including mild reduction in vibration sense in 3 (2.4%).

MRI signs of cSVD included WMH Fazekas stage 2 (n = 30) in 24.2% and Fazekas stage 3 (n = 94) in 75.8% of patients; lacunes and cerebral microbleeds were observed in 66.1% and 69.4% of patients, respectively.

A comparison of cSVD patients with controls according to the parameters of the Tinetti and 6-MWT tests is presented in Table 4.

According to the Tinetti test, patients with cSVD showed significant differences compared to controls in both the “balance” and “gait” subscales. Based on the combined score from both subscales, fall risk was classified as moderate in 34 patients (27.4%) and high in 18 patients (14.5%).

In the 6-MWT, gait parameters in cSVD patients compared with controls were characterized by a significant decrease in step length and an increase in base width, reduced gait speed, and an increase in both walking time and the number of steps required to cover the 6 m distance.

The relations between the results of the Tinetti and 6-MWT tests were clarified. Predominantly high or moderate statistically significant correlations were revealed.

Scores on the “balance” and “gait” subscales showed direct correlations with gait speed and maximum step length, and inverse correlations with walking time and number of steps over 6 m, as well as maximum base width.

For the parameters of 6-MWT that showed statistically significant correlations with the ones of the Tinneti test, ROC analysis was performed to clarify the diagnostic value of the parameters with respect to GD in cSVD. The results are presented in Table 5. All of them showed an area under the curve > |8|.

To assess the value of the Clinical Scale for Assessing the Severity of GDs in cSVD (RCN, 2019), groups of patients with varying severity of GD were compared with the results of the Tinetti and 6 m walk tests. The results are presented in Table 6. The small number of patients with profound GD (4 patients—3.2%) limited statistical comparison, and they were therefore excluded from the analysis. With increasing GD severity (according to Clinical Scale for Assessing the Severity of Gait Disorders in SVD (RCN, 2019)), there was a statistically significant deterioration in Tinetti test results (total score and “balance” and “gait” subscales), as well as in all measured gait parameters in the 6 m walk test.

Additionally, a comparison was performed between patients with predominantly frontal-cerebellar and frontal-subcortical types of severe GD, using Tinetti and 6 m walk tests parameters. The statistically significant results are presented in Table 7.

Patients with the frontal-subcortical type demonstrated significantly poorer results in the Tinetti test (“gait” subscale and total score) and in the 6-MWT (number of steps to cover 6 m and mean step length). The subgroups did not differ significantly in walking time over 6 m, maximum and minimum step length, base width, or gait speed.

## 4. Discussion

According to the results of this study of cSVD patients, the prevalence of GD of varying severity was 70%, which is generally consistent with other reports indicating a prevalence of this clinical syndrome of 30–85% in cSVD patients [4,9,40]. Similarly to other studies, GDs were the second most frequent clinical presentation of cSVD after CI [2,9,11,12]. The presence of CI is etiopathogenetically closely associated with GDs and clarification of their relationship is an independent, actively developed problem [41,42,43].

Based on the total Tinetti score, a moderate or high risk of falls was identified in more than 40% of cSVD patients, which is consistent with previous studies and indicates a high risk of disability due to falls in this group of patients. For example, in a study by Koo et al. (2012) assessing elderly individuals with WMH, the risk of falls, without specifying degree of probability, was present in 37.6% of participants (Tinetti score ≤ 24) [19]. Similar findings were reported by Zhao et al. (2022) in a study of 139 cSVD patients, where Tinetti score < 24 was found in 45.3% [44].

Comparison of gait parameters between cSVD patients and the control group showed that cSVD is characterized by reduced gait speed, shorter step length, and altered base width, in agreement with previous findings [12], and consistent with the qualitative gait characteristics described in the Clinical Scale for Assessing the Severity of GD in cSVD (RCN, 2019). Discrepancies with other studies were found in reference values for normal gait parameters [45,46,47,48,49,50,51]. In our study, the threshold gait speed for diagnosing GD was 0.75 m/s, which is slightly lower than in an earlier study [46] and comparable to the value reported by Li et al. [52].

In this cross-sectional study on a single group of cSVD patients, GDs were assessed using scales previously employed in international clinical studies on cSVD and with the highest citation rates—namely, the performance-based Tinetti test and the 6-MWT (with assessment of basic gait parameters) [53]—as well as the Clinical Scale for Assessing the Severity of GDs in cSVD (RCN, 2019). The aim was to clarify the diagnostic value, validity, and advantages of different scales in the assessment of GDs in cSVD patients in clinical practice. To date, no direct comparison of gait assessment scales in cSVD, including in published meta-analyses, has been performed [28,29,30].

Statistically significant, predominantly strong correlations were found between the results of the gait assessment using the Tinetti test (both the “balance” and “gait” subscales, as well as the total score characterizing fall risk) and the gait parameters obtained from the 6-MWT (direct correlations with maximum step length and gait speed and inverse correlations with time and number of steps), as well as threshold values of the latter (ROC analysis: area under the curve > 0.82) for diagnosing GDs in cSVD. These findings confirm the high diagnostic value of both scales for assessing GDs in cSVD. The advantage of the Tinetti test lies in its ability to assess the risk of falls based on the combined score of the “balance” and “gait” subscales, while the 6-MWT allows for the diagnosis of GD using threshold values for gait parameters (speed, time and number of steps over 6 m, and maximum step length) and their application for longitudinal monitoring.

Within the present study, results from the internationally recognized Tinetti test and the 6-MWT, which provide quantitative data, were compared with the Clinical Scale for Assessing the Severity of GDs in cSVD (RCN, 2019), which classifies GDs by severity categories. This scale was developed in 2019 based on clinical observation of a large cohort of cSVD patients. The validity of the proposed severity categories was confirmed by phase-contrast MRI [27]. Statistically significant associations were found between increasing GD severity and a growing imbalance in the brain’s fluid compartments, manifested as increased arterial stiffness with impaired compliance as well as reduced venous outflow and consequent elevation of venous pressure, leading to altered cerebrospinal fluid dynamics and progression of internal hydrocephalus (enlargement of the aqueduct and lateral ventricles) [27]. Such imbalance in cerebral homeostasis parameters is one of the most important pathogenetic mechanisms driving cSVD progression, and interest in elucidating its role in the development of cSVD and its clinical manifestations has markedly increased in recent years [54,55,56,57,58,59,60,61].

For patients in higher-severity categories of GDs, statistically significant intergroup differences (*p* < 0.001) were identified in declining Tinetti test results (total score, “balance” and “gait” subscales) and in all measured gait parameters of the 6-MWT. The observed correspondence between the defined severity categories of GDs and the parameters obtained from the Tinetti test and the 6-MWT supports the applicability of using this categorical scale for GD assessment in SVD. The main advantages of this scale are its simplicity, ease of administration, and the ability to determine the severity category in minimal time without additional tests or equipment, making it free from limitations for use in clinical practice with cSVD patients. Furthermore, the scale’s categories align with disease severity progression, enabling their use in research on the pathophysiological mechanisms of progressive GDs in cSVD and their relationship to microstructural changes in tracts involved in gait control.

An additional advantage of the scale is its ability to identify clinical subtypes of profound GDs that differ in risk of falls. Patients, clinically verified as having the frontal-subcortical subtype, demonstrated a statistically significant reduction in step length and, consequently, a greater number of steps in the 6-MWT, as well as a lower total score on the Tinetti test, which is associated with a higher risk of falls (compared with the frontal-cerebellar subtype). In some cases, distinguishing the exact phenotype is challenging. However, we believe that even identifying a predominance of the frontal-subcortical over the frontal-cerebellar pattern points to a predictably higher risk of falls and, accordingly, greater risk of disease-related complications. Nevertheless, these conclusions should be interpreted with caution given the small sample size of patients with pronounced GD in general, and of those with the frontal-cerebellar and frontal-subcortical subtypes in particular. Furthermore, the exclusion of participants with profound gait disorders may represent a limitation of the study.

In the present study, our aim was to compare well-established clinical scales, clarifying their value and advantages for assessing GDs in cSVD patients in clinical practice. Our data indicate strong interrelationships between scale parameters for gait assessment in cSVD patients. The advantages of the Tinetti test include the ability to assess the risk of falls, while the 6-MWT enables diagnosis of GDs based on threshold parameter values, which is particularly important in the early stages of the disease and for monitoring its progression. The Clinical Scale for Assessing the Severity of GDs in cSVD, along with its defined categories, can be applied in clinical practice as a simple and accessible screening tool to evaluate the GD and disease overall severity. It is especially relevant for identifying cSVD patients at high fall risk, particularly in the stage of pronounced GD and most notably in those with the frontal-subcortical subtype. Moreover, it can be used in research to study mechanisms of brain damage at different stages of cSVD and its relationship to specific patterns of CI.

## Figures and Tables

**Table 1:** Clinical Scale for Assessing the Severity of GDs in cSVD (RCN, 2019).

Severity of GD		Clinical Characteristics of Gait
0	No GD	No disorders, including in complicated samples (phalanx, tandem walking)
1	Mild	Instability when performing the complicated samples
2	Moderate	Shortening of the length of the step, a slowing down of the rate, wherein the rhythm and base of the support correspond to the norm
3	Severe	Shortening of the length of the pitch relative to the norm, difficulties in turning, but with retention of walking without support
3a		A—frontal-cerebellar type: the presence of an increase in the base of the support relative to the norm, “adhesion” of the feet to the floor during walking and instability during flanking and tandem walking, wherein there is no posture change, disturbed walking, and propulsion initiation
3b		B—frontal-subcortical type: shortening of length of pitch and support base reduction in standing position relative to norm, presence of disturbed initiation of walking in form of obstruction of walking, change in posture, accompanied by forward inclination, and presence of propulsions
4	Profound	The need for single- or double-sided support

**Table 2:** General characteristics of patients with cSVD and control group.

Parameter	cSVD (n = 124)	Control (n = 30)	*p*
Sex, n (%)			0.734
male	58 (46.8%)	13 (43.3%)	
female	66 (53.2%)	17 (56.7%)	
Age, years (M ± SD)	62.18 ± 7.852	59.77 ± 6.361	0.121
HTN, n (%)	122 (98.4%)	17 (56.7%)	*p* < 0.05
HTN stage, n (%)			
1	11 (8.9%)	8 (26.7%)	
2	35 (28.2%)	4 (13.3%)	
3	76 (61.3%)	5 (16.7%)	
Type 2 diabetes mellitus, n (%)	27 (21.8%)	1 (3.3%)	*p* < 0.05
Cholesterol mmol/ L (Me [25%; 75%])	5.60 [4.70; 6.70]	5.20 [4.10; 6.10]	*p* < 0.05
Obesity, n (%)	50 (40.3%)	6 (20.0%)	*p* < 0.05
Smoking, n (%)	39 (31.5%)	10 (33.3%)	0.976

**Table 3:** General characteristic of cSVD patients.

Parameter	cSVD (n = 124)
CI, n (%)	124 (100%)
Subjective/Moderate/Dementia	44 (35.5%)/47 (37.9%)/33 (26.6%)
MoCA score (M ± SD)	22.85 ± 4.72
GD, n (%)	85 (68.5%)
Mild	34 (27.4%)
Moderate	18 (14.5%)
Severe	29 (23.4%)
Profound	4 (3.2%)
Pseudobulbar palsy, n (%)	35 (28.2%)
Urinary disturbances, n (%)	38 (30.6%)
Sensory polyneuropathy, n (%)	8 (6.5%)
Mild pallhypesthesia, n (%)	3 (2.4%)
Proprioceptive deficit, n (%)	0 (0%)
Arthrosis of the joints of the lower limbs 1–2 grade, n (%)	4 (3.2%)
White matter hyperintensity, Fazekas scale, n (%)	
F2/F3	30 (24.2%)/94 (75.8%)
Lacunes, n (%)	82 (66.1%)
Microbleeds, n (%)	86 (69.4%)
Enlarged perivascular spaces, n (%)	123 (99.2%)

**Table 4:** Characteristics of the Tinetti test and gait parameters in the “6-m walk” test in patients with cSVD and in the control group.

Parameter Me [25%; 75%]	cSVD (n = 124)	Control (n = 30)	*p*
Tinetti Test			
Balance score	14.0 [11.0; 15.0]	16.0 [16.0; 16.0]	<0.05
Gait score	11.0 [10.0; 12.0]	12.0 [12.0; 12.0]	<0.05
Total Test score	25.0 [21.0; 27.0]	28.0 [28.0; 28.0]	<0.05
Risk of falls, n (%)			
Low	72 (58.1%)	30 (100%)	<0.05
Medium	34 (27.4%)		
High	18 (14.5%)		
“6-m Walk” Test			
Total time taken to ambulate 6 m, s	8.50 [6.80; 10.89]	5.50 [4.90; 6.20]	<0.05
Number of steps in “6-m walk” test, n	13.00 [11.00; 16.00]	9.00 [9.00; 10.00]	<0.05
Maximum step length, cm	55.00 [46.00; 64.45]	70.25 [63.00; 77.00]	<0.05
Minimum step length, cm	47.00 [35.50; 54.00]	64.25 [57.00; 71.50]	<0.05
Maximum base width, cm	31.00 [26.15; 35.75]	23.00 [19.60; 27.00]	<0.05
Minimum base width, cm	23.50 [19.25; 30.00]	16.00 [13.50; 18.50]	<0.05
Gait speed, m/s	0.71 [0.55; 0.88]	1.09 [0.97; 1.22]	<0.05

**Table 5:** Thresholds and characteristics of the area under the curve of parameters in the “6-m walk” test for GD diagnosis in cSVD according to ROC analysis.

Parameter	Threshold Value	Sensitivity, %	Specificity, %	AUC	95% Confidence Interval		*p*
Gait speed, m/s	0.75	79.4%	72.0%	0.842	0.780	0.905	<0.001
Total time taken to ambulate 6 m, s	7.24	80.5%	70.6%	0.842	0.780	0.905	<0.001
Number of steps in “6-m walk” test, n	11.5	80.5%	72.1%	0.844	0.782	0.906	<0.001
Maximum step length, cm	56.5	82.4%	72.0%	0.820	0.754	0.886	<0.001
Maximum base width, cm	28.6	78.0%	73.5%	0.812	0.740	0.876	<0.001

**Table 6:** Comparative analysis of Tinetti test and gait parameters in “6-m walk” test between groups of patients with varying GD severity according to the Clinical Scale for Assessing the Severity of GDs in cSVD.

Parameter Me [25%; 75%]	No Gait Disorders 0 (n = 39)	Mild 1 (n = 34)	Moderate 2 (n = 18)	Severe 3 (n = 29)	*p*
Tinetti Test					
Balance score	16.0 [15.0; 16.0]	14.0 [12.75; 15.25]	12.0 [10.5; 14.0]	10.0 [8.0; 11.0]	*p*_0-2,3; 1-3_ < 0.001 *p*_1-0_ = 0.003
Gait score	12.0 [12.0; 12.0]	12.0 [11.0; 12.0]	11.0 [10.5; 12.0]	8.0 [7.0; 10.0]	*p*_3-0,1_ < 0.001 *p*_3-2_ = 0.001 *p*_2-0_ = 0.007
Total test score	28.0 [27.0; 28.0]	26.0 [24.0; 27.0]	23.0 [21.0; 25.0]	18.5 [15.0; 21.0]	*p*_3-0,1; 2-0_ < 0.001 *p*_1-0_ = 0.001
“6-m Walk” Test					
Total time taken to ambulate 6 m, s	6.08 [5.41; 7.8]	8.36 [6.78; 9.14]	8.5 [7.25; 10.18]	13.36 [9.83; 16.2]	*p*_0-3_ < 0.001 *p*_1-3_ = 0.001 *p*_0-1,2_ = 0.002
Number of steps in “6-m walk” test, n	10.0 [9.0; 12.0]	12.5 [10.75; 14.0]	13.0 [12.0; 15.0]	18.0 [15.0; 23.25]	*p*_0-2,3; 1-3_ < 0.001 *p*_0-1_ = 0.005
Maximum step length, cm	66.0 [59.0; 73.0]	54.0 [52.0; 67.63]	54.0 [47.0; 58.0]	41.25 [37.0; 51.75]	*p*_0-3_ < 0.001 *p*_3-1;2-0_ = 0.001 *p*_1-0_ < 0.05
Gait speed, m/s	0.99 [0.77; 1.11]	0.72 [0.66; 0.89]	0.71 [0.59; 0.83]	0.45 [0.37; 0.61]	*p*_3-0_ < 0.001 *p*_3-1_ = 0.001 *p*_2-0;1-0_ = 0.002

**Table 7:** Comparative analysis of Tinetti test and gait parameters in “6-m walk” test in patients with frontal-cerebellar and frontal-subcortical types of GD.

Parameter	Frontal-Cerebellar Type (n = 11)	Frontal-Subcortical Type (n = 18)	*p*
Tinetti Test			
Balance score	11.0 [11.0; 12.0]	9.0 [8.0; 11.0]	0.057
Gait score	10.0 [8.0; 11.0]	8.0 [7.0; 9.0]	0.031
Total test score	21.0 [18.0; 22.0]	17.0 [15.0; 20.0]	0.031
Results of individual tests in subscales:			
*Arises, n (%):* ◊ unable without help ◊ able, uses arms to help ◊ able without using arms	0 (0%) 4 (36.4%) 6 (54.5%)	2 (11.1%) 14 (77.8%) 2 (11.1%)	0.019
*Attempts to Arise, n (%):* ◊ unable without help ◊ able, requires > 1 attempt ◊ able to rise, 1 attempt	0 (0%) 1 (9.1%) 9 (81.8%)	0 (0%) 11 (61.1%) 7 (38.9%)	0.009
*Right swing foot, n (%):* ◊ does not pass left stance foot with step ◊ does not pass left stance foot with step	1 (9.1%) 9 (81.8%)	11 (61.1%) 7 (38.9%)	0.009
“6-m Walk” Test			
Number of steps in “6-m walk” test, n	15.00	20.00	0.035
	[13.00; 18.00]	[18.00; 24.00]	
Average step length, cm (based on the number of steps per 6 m)	40.00	30.79	0.035
	[33.33; 46.15]	[25.00; 33.33]	

## Data Availability

The data presented in this study are available upon reasonable request from the corresponding author.

## Abbreviations

The following abbreviations are used in this manuscript:

cSVD	cerebral small vessel disease
CI	cognitive impairment
GDs	gait disorders
MCI	mild cognitive impairment
subCI	subjective cognitive impairment
MRI	magnetic resonance imaging

## References

[1] Pantoni L. (2010). Cerebral small vessel disease: From pathogenesis and clinical characteristics to therapeutic challenges. Lancet Neurol..

[2] de Leeuw F.E., de Groot J.C., Achten E., Oudkerk M., Ramos L.M., Heijboer R., Hofman A., Jolles J., van Gijn J., Breteler M.M. (2001). Prevalence of cerebral white matter lesions in elderly people: A population based magnetic resonance imaging study. The Rotterdam Scan Study. J. Neurol. Neurosurg. Psychiatry.

[3] Wardlaw J.M., Smith E.E., Biessels G.J., Cordonnier C., Fazekas F., Frayne R., Lindley R.I., O’Brien J.T., Barkhof F., Benavente O.R. (2013). Neuroimaging standards for research into small vessel disease and its contribution to ageing and neurodegeneration. Lancet Neurol..

[4] Stolze H., Klebe S., Baecker C., Zechlin C., Friege L., Pohle S., Deuschl G. (2005). Prevalence of gait disorders in hospitalized neurological patients. Mov. Disord..

[5] de Laat K.F., van Norden A.G., Gons R.A., van Oudheusden L.J., van Uden I.W., Bloem B.R., Zwiers M.P., de Leeuw F.E. (2010). Gait in elderly with cerebral small vessel disease. Stroke.

[6] Srikanth V., Phan T.G., Chen J., Beare R., Stapleton J.M., Reutens D.C. (2010). The location of white matter lesions and gait—A voxel-based study. Ann. Neurol..

[7] Pirker W., Katzenschlager R. (2017). Gait disorders in adults and the elderly: A clinical guide. Wien. Klin. Wochenschr..

[8] Ataullah A.H.M., De Jesus O. (2024). Gait Disturbances. StatPearls.

[9] Okroglic S., Widmann C.N., Urbach H., Scheltens P., Heneka M.T. (2013). Clinical symptoms and risk factors in cerebral microangiopathy patients. PLoS ONE.

[10] Pinter D., Ritchie S.J., Doubal F., Gattringer T., Morris Z., Bastin M.E., Del C Valdés Hernández M., Royle N.A., Corley J., Muñoz Maniega S. (2017). Impact of small vessel disease in the brain on gait and balance. Sci. Rep..

[11] Su C., Yang X., Wei S., Zhao R. (2022). Association of Cerebral Small Vessel Disease with Gait and Balance Disorders. Front. Aging Neurosci.

[12] van der Holst H.M., van Uden I.W., Tuladhar A.M., de Laat K.F., van Norden A.G., Norris D.G., van Dijk E.J., Rutten-Jacobs L.C., de Leeuw F.E. (2016). Factors Associated With 8-Year Mortality in Older Patients with Cerebral Small Vessel Disease: The Radboud University Nijmegen Diffusion Tensor and Magnetic Resonance Cohort (RUN DMC) Study. JAMA Neurol..

[13] Brissaud E. (1895). Leçons sur les Maladies Nerveuses.

[14] Binswanger O. (1894). Die abgrenzung der allgemeinen progressiven paralyse. Berl. Klin. Wochenschr..

[15] Baezner H., Hennerici M. (2005). From trepidant abasia to motor network failure--gait disorders as a consequence of subcortical vascular encephalopathy (SVE): Review of historical and contemporary concepts. J. Neurol. Sci..

[16] Baezner H., Hennerici M., Pantoni L., Gorelick P.B. (2014). Cerebral Small Vessel Disease.

[17] Xu K., Wang Y., Jiang Y., Wang Y., Li P., Lu H., Suo C., Yuan Z., Yang Q., Dong Q. (2024). Analysis of gait pattern related to high cerebral small vessel disease burden using quantitative gait data from wearable sensors. Comput. Methods Programs Biomed..

[18] Tinetti M.E. (1986). Performance-oriented assessment of mobility problems in elderly patients. J. Am. Geriatr. Soc..

[19] Koo B.B., Bergethon P., Qiu W.Q., Scott T., Hussain M., Rosenberg I., Caplan L.R., Bhadelia R.A. (2012). Clinical prediction of fall risk and white matter abnormalities: A diffusion tensor imaging study. Arch. Neurol..

[20] Zhang W., Shen H., Yao X., Liu F., Wang S., Yang Y., Zhang N., Wang C. (2020). Clinical and Diffusion Tensor Imaging to Evaluate Falls, Balance and Gait Dysfunction in Leukoaraiosis: An Observational, Prospective Cohort Study. J. Geriatr. Psychiatry Neurol..

[21] Hou Y., Li Y., Yang S., Qin W., Yang L., Hu W. (2021). Gait impairment and upper extremity disturbance are associated with Total magnetic resonance imaging cerebral small vessel disease burden. Front. Aging Neurosci..

[22] Pinter D., Ritchie S.J., Gattringer T., Bastin M.E., Hernández M.D.C.V., Corley J., Maniega S.M., Pattie A., Dickie D.A., Gow A.J. (2018). Predictors of gait speed and its change over three years in community-dwelling older people. Aging (Albany NY).

[23] Heiland E.G., Welmer A.K., Kalpouzos G., Laveskog A., Wang R., Qiu C. (2021). Cerebral small vessel disease, cardiovascular risk factors, and future gait speed in old age: A population-based cohort study. BMC Neurol..

[24] Wang L., Lin H., Peng Y., Zhao Z., Chen L., Wu L., Liu T., Li J., Liu A., Lo C.-Y.Z. (2021). Incidental Brain Magnetic Resonance Imaging Findings and the Cognitive and Motor Performance in the Elderly: The Shanghai Changfeng Study. Front. Neurosci..

[25] Su N., Mao H.J., Zhang J., Zhu W.-C., Han F., Yao M., Zhou L.-X., Ni J., Fan X.-M., Tian F. (2023). Analysis of clinical and imaging characteristics of patients with freezing of gait in cerebral small vessel disease (P11-5.004). Neurology.

[26] Jokinen H., Laakso H.M., Ahlström M., Arola A., Lempiäinen J., Pitkänen J., Paajanen T., Sikkes S.A.M., Koikkalainen J., Lötjönen J. (2022). Synergistic associations of cognitive and motor impairments with functional outcome in covert cerebral small vessel disease. Eur. J. Neurol..

[27] Dobrynina L., Bitsieva E., Byrochkina A., Tsypushtanova M., Makarova A., Zabitova M., Shamtieva K., Akhmetshina Y., Trubitsyna V. (2024). Gait disorders in cerebral small vessel disease: The role of cerebrospinal fluid flow and venous drainage impairment. Eur. J. Neurol..

[28] Gor-García-Fogeda M.D., Cano de la Cuerda R., Carratalá Tejada M., Alguacil-Diego I.M., Molina-Rueda F. (2016). Observational Gait Assessments in People with Neurological Disorders: A Systematic Review. Arch. Phys. Med. Rehabil..

[29] Ridao-Fernández C., Pinero-Pinto E., Chamorro-Moriana G. (2019). Observational Gait Assessment Scales in Patients with Walking Disorders: Systematic Review. Biomed. Res. Int..

[30] Beck Jepsen D., Robinson K., Ogliari G., Montero-Odasso M., Kamkar N., Ryg J., Freiberger E., Masud T. (2022). Predicting falls in older adults: An umbrella review of instruments assessing gait, balance, and functional mobility. BMC Geriatr..

[31] Christopher A., Kraft E., Olenick H., Kiesling R., Doty A. (2021). The reliability and validity of the Timed Up and Go as a clinical tool in individuals with and without disabilities across a lifespan: A systematic review. Disabil. Rehabil..

[32] Miranda N., Tiu T.K. (2023). Berg Balance Testing. StatPearls.

[33] Santamaría-Peláez M., González-Bernal J.J., Da Silva-González Á., Medina-Pascual E., Gentil-Gutiérrez A., Fernández-Solana J., Mielgo-Ayuso J., González-Santos J. (2023). Validity and Reliability of the Short Physical Performance Battery Tool in Institutionalized Spanish Older Adults. Nurs. Rep..

[34] Nasreddine Z.S., Phillips N.A., Bédirian V., Charbonneau S., Whitehead V., Collin I., Cummings J.L., Chertkow H. (2005). The Montreal Cognitive Assessment, MoCA: A brief screening tool for mild cognitive impairment. J Am. Geriatr. Soc..

[35] Mahoney F.I., Barthel D.W. (1965). Functional evaluation: The Barthel index. Md. State Med. J..

[36] Lawton M.P., Brody E.M. (1969). Assessment of older people: Self-maintaining and instrumental activities of daily living. Gerontologist.

[37] Lewis C. (1993). Balance, gait test proves simple yet useful. PT Bull..

[38] Tiedemann A., Shimada H., Sherrington C., Murray S., Lord S. (2008). The comparative ability of eight functional mobility tests for predicting falls in community-dwelling older people. Age Ageing.

[39] Skvortsov D.V. (1996). Clinical analysis of movements. Gait analysis.

[40] Baezner H., Blahak C., Poggesi A., Pantoni L., Inzitari D., Chabriat H., Erkinjuntti T., Fazekas F., Ferro J.M., Langhorne P. (2008). Association of gait and balance disorders with age-related white matter changes: The LADIS study. Neurology.

[41] Feng M., Song Z., Zhou Z., Wu Z., Ma M., Liu Y., Wang Y., Dai H. (2024). Cognitive impairment mediates the white matter injury load and gait disorders in subcortical ischemic vascular disease. Brain Imaging Behav..

[42] Li H., Jacob M.A., Cai M., Kessels R.P.C., Norris D.G., Duering M., de Leeuw F.-E., Tuladhar A.M. (2024). Meso-cortical pathway damage in cognition, apathy and gait in cerebral small vessel disease. Brain.

[43] Liu Y., Zhang L., Yang S., Liu R., Yi L., Liu M., Liu S., Zhang Z. (2025). Predictive role of gait parameters and MRI markers in assessing cognitive decline in CSVD patients. BMC Geriatr..

[44] Zhao P., Gu Y., Feng W., Xia X., Tian X., Du Y., Li X. (2022). Gait Disorders and Magnetic Resonance Imaging Characteristics in Older Adults with Cerebral Small Vessel Disease. J. Integr. Neurosci..

[45] Oberg T., Karsznia A., Oberg K. (1993). Basic gait parameters: Reference data for normal subjects, 10-79 years of age. J. Rehabil. Res. Dev..

[46] Samson M.M., Crowe A., de Vreede P.L., Dessens J.A., Duursma S.A., Verhaar H.J. (2001). Differences in gait parameters at a preferred gait speed in healthy subjects due to age, height and body weight. Aging (Milano).

[47] Cesari M., Kritchevsky S.B., Penninx B.W., Nicklas B.J., Simonsick E.M., Newman A.B., Tylavsky F.A., Brach J.S., Satterfield S., Bauer D.C. (2005). Prognostic value of usual gait speed in well-functioning older people--results from the Health, Aging and Body Composition Study. J. Am. Geriatr. Soc..

[48] Oh-Park M., Holtzer R., Xue X., Verghese J. (2010). Conventional and robust quantitative gait norms in community-dwelling older adults. J. Am. Geriatr. Soc..

[49] Hollman J.H., McDade E.M., Petersen R.C. (2011). Normative spatiotemporal gait parameters in older adults. Gait Posture.

[50] Hass C.J., Malczak P., Nocera J., Stegemöller E.L., Wagle S. A., Malaty I., Jacobson C.E., Okun M.S., McFarland N. (2012). Quantitative normative gait data in a large cohort of ambulatory persons with Parkinson’s disease. PLoS ONE.

[51] Middleton A., Fritz S.L., Lusardi M. (2015). Gait speed: The functional vital sign. J. Aging Phys. Act..

[52] Li P., Wang Y., Jiang Y., Zhang K., Yang Q., Yuan Z., Zhu Z., Tang W., Fan M., Ye W. (2020). Cerebral small vessel disease is associated with gait disturbance among community-dwelling elderly individuals: The Taizhou imaging study. Aging.

[53] Sharma B., Wang M., McCreary C.R., Camicioli R., Smith E. (2023). Gait and falls in cerebral small vessel disease: A systematic review and meta-analysis. Age Ageing.

[54] Dobrynina L.A., Akhmetzyanov B.M., Gadzhieva Z.S., Kremneva E.I., Kalashnikova L.A., Krotenkova M.V. (2019). The role of arterial and venous blood flow and cerebrospinal fluid flow disturbances in the development of cognitive impairments in cerebral microangiopathy. Ann. Clin. Exp. Neurol..

[55] Dobrynina L.A., Gadzhieva Z.S., Shamtieva K.V., Kremneva E.I., Akhmetzyanov B.M., Tsypushtanova M.M., Makarova A.G., Trubitsyna V.V., Krotenkova M.V. (2022). Relations of impaired blood flow and cerebrospinal fluid flow with damage of strategic for cognitive impairment brain regiones in cerebral small vessel disease. Ann. Clin. Exp. Neurol..

[56] Blair G.W., Thrippleton M.J., Shi Y., Hamilton I., Stringer M., Chappell F., Dickie D.A., Andrews P., Marshall I., Doubal F.N. (2020). Intracranial hemodynamic relationships in patients with cerebral small vessel disease. Neurology.

[57] Birnefeld J., Wåhlin A., Eklund A., Malm J. (2020). Cerebral arterial pulsatility is associated with features of small vessel disease in patients with acute stroke and TIA: A 4D flow MRI study. J. Neurol..

[58] Zhang R., Huang P., Jiaerken Y., Wang S., Hong H., Luo X., Xu X., Yu X., Li K., Zeng Q. (2021). Venous disruption affects white matter integrity through increased interstitial fluid in cerebral small vessel disease. J. Cereb. Blood Flow Metab..

[59] Binnie L.R., Pauls M.M.H., Benjamin P., Dhillon M.K., Betteridge S., Clarke B., Ghatala R., Hainsworth F.A.H., Howe F.A., Khan U. (2022). Test-retest reliability of arterial spin labelling for cerebral blood flow in older adults with small vessel disease. Transl. Stroke Res..

[60] Webb A.J.S., Werring D.J. (2022). New Insights into Cerebrovascular Pathophysiology and Hypertension. Stroke.

[61] Morgan A.G., Thrippleton M.J., Stringer M., Jin N., Wardlaw J.M., Marshall I. (2023). Repeatability and comparison of 2D and 4D flow MRI measurement of intracranial blood flow and pulsatility in healthy individuals and patients with cerebral small vessel disease. Front. Psychol..

